# A study on the reasonable dietary trajectory of elderly people in the community and its correlation with body mass index

**DOI:** 10.3389/fnut.2024.1429627

**Published:** 2024-08-01

**Authors:** Mengya Liu, Yuqing Chang, Wenjing Guo, Siyi Zhao, Meng Zhang, Xiaoyan Ma, Xiaomei Ji, Youran Liu, Li Zhang

**Affiliations:** Bengbu Medical University School of Nursing Bengbu, Bengbu, China

**Keywords:** reasonable diet, body mass index, older adults, aging, China

## Abstract

**Objective:**

To explore the reasonable dietary trajectory of elderly people in the community and to test the correlation between different dietary trajectories and body mass index (BMI) of the elderly people in the community to provide a reference for these individuals to formulate scientific interventions and cultivate healthy living habits.

**Methods:**

The data of The Chinese Longitudinal Healthy Longevity Survey (CLHLS) from 2011 to 2018 were used to evaluate the dietary status of elderly people in the community according to their diet, and body mass index was calculated according to height and weight. The latent variable growth mixed (LGMM) model was used to analyze the development trajectory of diet in elderly people, and the multivariate logistic regression model was used to test the relationship between different dietary development trajectories and BMI changes as well as to test the correlation between different dietary trajectories and BMI of the elderly people in the community.

**Results:**

The LGMM fit four dietary trajectories of elderly individuals: the continuous reasonable diet group (37.81%), the dietary behavior decline group (28.84%), the continuous unreasonable diet group (20.16%), and the dietary behavior improvement group (13.19%). The results showed that factors including male sex, rural setting, being spouseless, nonformal education status, not being wealthy, living alone, and having tooth loss were more likely to be classified as the “persistently unreasonable diet group” (*p* < 0.05). The logistic regression results showed that the “continuous reasonable diet group” and the “dietary behavior improvement group” were significantly correlated with the development of obesity to a normal BMI.

**Conclusion:**

The dietary behavior of the elderly was significantly correlated with BMI value, and improving the reasonable dietary behavior of the elderly could reduce the high BMI to the normal range, but could not restore the low BMI to the normal range, indicating that reasonable dietary behavior is an important measure to prevent and improve overweight or obesity in the elderly. There is significant heterogeneity in the dietary behavior of the elderly, and community medical staff should identify the risk factors of various dietary behaviors of other groups as soon as possible, and provide corresponding intervention strategies to help them change their poor dietary behaviors and improve their nutritional status.

## 1 Introduction

According to the National Bureau of Statistics, by the end of 2022, there were approximately 210 million elderly people aged 65 years and above in China, accounting for 14.9% of the total population; this result demonstrates that China has become a deep aging society (1). The greatest challenge posed by the aging population is the severe and complex health problems of elderly people. According to the Report on Nutrition and Health of the Elderly in China, there is a twofold burden of malnutrition and overnutrition among elderly people in China (2). With age, the functions of various body systems gradually decline; moreover, nutritional knowledge is lacking, and insufficient intake of energy, protein and other nutrients leads to an imbalance in the body’s needs and intake, thus increasing the risk of malnutrition. According to previous studies, 48.4% of elderly individuals in China have poor nutritional status, and the overall prevalence of anemia among elderly individuals can reach 10.6% (3). Furthermore, due to rapid socioeconomic development, the quality of life of elderly people has improved, and some elderly people exhibit excessive fat, sugar or salt intake and perform less physical labor, thus resulting in an obvious trend toward overnutrition and an increase in the number of overweight and obese individuals. According to the Fifth National Physical Fitness Monitoring Bulletin, the overweight and obesity rates of elderly people in China are 41.7 and 16.7%, respectively (4).

A reasonable diet is the cornerstone of ensuring the health of elderly people, and it is closely related to the maintenance of physical function and quality of life. In 2022, the Chinese Nutrition Society released the Dietary Guidelines for the Elderly (2022), which includes two parts: the general elderly population, aged 65–79 years, and the elderly population, aged 80 years and above (5). In general, older people have a lower need for energy with age, but their need for important nutrients (such as protein) increases. However, due to the decline of sensory functions such as taste, the choice of food gradually solidifies with age, thus resulting in a single variety of food being consumed. The guidelines suggest that for the general elderly population, it is necessary to ensure that there is a variety of food, maintain a good appetite and appropriate weight, and prevent nutritional deficiencies (5). Elderly people often have limited food intake for various reasons, and their demand for energy and nutrients increases. The guidelines suggest that for elderly people, it is necessary to ensure food diversity; moreover, they should eat more fish, poultry, eggs, milk and beans, as well as undergo regular nutritional screenings and receive timely nutritional supplementations (5). These guidelines can help elderly people to better adapt to changes in physical function, and a reasonable diet plays an important role in preventing and delaying the occurrence and development of disease, thus prolonging a healthy lifespan, improving quality of life, and promoting healthy aging.

According to the Report on Nutrition and Health of the Elderly in China, unbalanced dietary intake is the main reason for the dual burden of malnutrition and overnutrition among elderly people (2). Body mass index (BMI) is one of the most commonly used indicators in clinical practice to assess malnutrition or overnutrition in a population; additionally, BMI can not only determine the level of obesity in individuals but can also be used to preliminarily screen people who may be at risk of malnutrition. BMI is the standard for defining the degree of obesity and thinness of individuals, as well as whether they are healthy (6); furthermore, the range of BMI for normal adults is 18.5 kg/m^2^–23.9 kg/m^2^ (7). In 1997, the World Health Organization (WHO) first recognized obesity as a disease and used body mass index (BMI) as a common measure of overweight/obesity (8). Overweight or obesity is an important manifestation of overnutrition in the elderly population, and there is no doubt that BMI is used to measure overnutrition in the elderly population internationally; moreover, being overweight or obese represent key risk factors for other disorders, such as cardiovascular disease, diabetes, stroke, and certain cancers, which seriously damage their physical and mental health (9). A low BMI is classified as a state of underweight, and Wang et al. (10) used a low BMI to investigate the incidence and trend of malnutrition among elderly individuals in China. Most foreign scholars have conducted clinical studies on the direct representation of low BMI as an indicator of malnutrition and found that it can predict a variety of adverse outcomes, such as frailty and death, thus confirming the clinical value of this indicator (11–13).

The elderly population is a unique group; specifically, there is considerable heterogeneity in this group, and their dietary behavior tends to show different trends due to the multifaceted effects of environmental and other social factors. Previous studies have focused on cross-sectional discussions on the impact of dietary patterns on a disease, ignoring changes in dietary behavior or structure throughout the life course. The BMI range of the elderly population is different from that of the young and middle-aged population, and the appropriate BMI range for the general elderly is 20.0 kg/m^2^–26.9 kg/m^2^ in the Dietary Guidelines for the Elderly, and “Appropriate range of body mass index and body weight management guidelines for Chinese oldest old” (13) clearly pointed out that the appropriate weight management target for the elderly is 22.0 kg/m^2^–26.9 kg/m^2^ to prevent the occurrence of adverse health outcomes and reduce or delay the occurrence of related diseases and their complications. Whether the BMI standard proposed in the guidelines is related to the reasonable dietary behavior of the elderly, that is, whether the change of reasonable diet can make the BMI of the elderly normal, and how the change between the two is a question that we need to explore. In order to solve the above problems, this study tested the data from the tracking survey of the influencing factors of the elderly health in China, used the latent variable growth mixed model (LGMM) to explore the dietary development trajectory of the elderly in the community, depicted the development trajectory curve of each subgroup, analyzed the influencing factors of different dietary trajectories of the elderly, and explored the longitudinal association between each trajectory group and BMI, so as to provide a reference for the elderly in the community to formulate scientific personalized interventions and cultivate healthy living habits.

## 2 Materials and methods

### 2.1 Research subjects

The Chinese Longitudinal Healthy Longevity Survey CLHLS is a longitudinal survey of elderly people organized by the Center for Healthy Aging and Development Research of Peking University/National Academy of Development, and it encompasses 23 provinces, municipalities and autonomous regions across the country, accounting for 85% of the national population. The survey subjects are elderly people aged 65 years and older and adult children aged 35–64 years. The questionnaire includes the basic status of elderly people and their families, socioeconomic background and family structure, economic resources and economic status, health and quality of life self-assessment, cognitive function, personality and psychological characteristics, daily activity ability, diet and other lifestyles, life care, and disease treatment, among other items. The survey was followed by a baseline survey in 1998 and follow-up surveys in 2000, 2002, 2005, 2008–2009, 2011–2012, 2014, and 2017–2018 (14). According to the CLHLS survey team, the study was approved by the Ethics Committee of Peking University (IRB00001052-13074), and all of the respondents signed an informed consent form before participating (15).

This study used demographic and health-related CLHLS data from 2011 to 2018, and a total of 9,765 respondents participated in the interviews in 2011. The inclusion criteria were as follows: (1) were aged ≥65 years, (2) had complete demographic data, required health-related data, and lifestyle data, among other information. The exclusion criteria: Respondents who did not participate in longitudinal tracking in the CLHLS. According to the inclusion and exclusion criteria, a total of 1,349 subjects participated in the 7-year follow-up.

### 2.2 Research method

#### 2.2.1 General information survey

By querying various books and electronic information databases related to the diet and BMI of elderly people at home and abroad, as well as investigating the research situation in various databases, such as books, journals, master’s and doctoral theses, newspapers and periodicals, the relevant variables were identified and sorted. The survey data that were collected included age, sex, education level, marital status, place of residence, economic status, smoking status, alcohol consumption status, cohabitation, exercise status, tooth loss status and the number and types of comorbidities.

#### 2.2.2 Dietary assessment

According to the Dietary Guidelines for the Elderly (2022) (5), elderly people should ensure their energy supply, maintain a daily grain intake of 200–300 g, control salt, sugar and oil intake, and eat more fresh vegetables and fruits as well as foods that are rich in important nutrients such as protein. Furthermore, ensuring food diversity is essential for an adequate and balanced nutritional supply. Therefore, this study selected 14 foods that are relevant to the dietary guidelines based on the CLHLS database and the dietary scoring methods of previous researchers, which is consistent with the principle of selecting and evaluating food groups for a reasonable diet according to a specific purpose (16, 17). The respondents were asked to report their intake and frequency of consumption of grains and oils, as well as 12 other food groups, including vegetables, fruits, meat, aquatic products, eggs, soy products, dairy products, pickles, sweets, fungi and algae, nuts, and vitamins.

Cereals and oils are foods that reflect the calories of elderly people, and a reasonable diet should be controlled within the normal range; for this reason, cereals and oils should be scored according to the amount and type of intake. “The amount of staple food per day is controlled at 200–300 g” was counted as 1 point, and “the amount of staple food per day is more than 300 g” and “the amount of staple food per day is less than 200 g” was counted as 0 points. “Mainly edible vegetable oil” was recorded as 1 point, and “regular consumption of animal oil such as lard” was recorded as 0 points. The other 12 foods were scored according to the frequency of intake, with participants responding to five of them (“almost every day,” “at least once a week,” “at least once a month,” “occasionally” and “rarely or never”), with 1 point being given if the answer to a food group was “almost every day” or “at least once a week”; otherwise, the score was 0. Moreover, pickled products and sweets were encouraged to be eaten less, and a reverse score was needed. The total score is the sum of the abovementioned 14 food scores, with a range of 0–14 points; a higher score indicated a more reasonable diet. The questionnaire was created to assess the adequacy of meals and the health of diets in older adults, and its scientific validity has been previously demonstrated (18). Based on the median total score, the respondents were divided into two groups: the reasonable diet group and the irreasonable diet group.

#### 2.2.3 Body mass index assessment

Body mass index (BMI) was calculated based on the height and weight measured in the questionnaire, and BMI was calculated by dividing the weight by the square of the height (m^2^) in kg/m^2^. Numerous studies (both in China and abroad) have shown that being too thin in elderly people can lead to reduced resistance and an increased risk of death (18, 19). According to the Dietary Guidelines for the Elderly, the BMI cutoff value for assessing whether elderly individuals have a normal weight is different from that of young and middle-aged people. The basic consensus formed by experts and scholars is that the weight of elderly individuals should not be too low, and a BMI of the general elderly is more suitable at 20.0 kg/m^2^–26.9 kg/m^2^ (5), the appropriate range of body mass index for Chinese oldest old is 22.0 kg/m^2^–26.9 kg/m^2^ (13).

### 2.3 Covariates

Data related to a reasonable diet and BMI were selected and coded, including age (65–79 years = 1, ≥80 years = 2), sex (male = 1, female = 2), marital status (with spouse = 1, without spouse = 2), education (below primary school = 1, primary school and above = 2), place of residence (rural = 1, urban = 2), economic status (low to moderate income = 1, high income = 2), smoking status (yes = 1, no = 2), alcohol consumption status (yes = 1, no = 2), living together (living with family = 1, living alone = 2, nursing home = 3), exercise status (yes = 1, no = 2), tooth loss status (yes = 1, no = 2), and comorbidities of chronic disease (yes = 1, no = 2).

### 2.4 Statistical methods

Normally distributed data are expressed as the mean ± standard deviation (x ± s), nonnormally distributed data are expressed as the median (M) and interquartile range (IQR), and counts are described as the frequency and percentage. The latent variable growth mixed model (LGMM) was used to fit the dietary trajectories of the elderly participants. LGMM combines the features of the linear mixed model with additional components to divide the population into a limited number of potential classes (20). The evaluation indicators of LGMM models include the Aickeck information criterion (AIC), Bayesian information criterion (BIC), sample-corrected BIC (aBIC), information entropy, likelihood ratio test (LMR), and bootstrap-based likelihood ratio test (BLRT) (21). Lower values of AIC, BIC, and aBIC indicated a better fit of the model. Moreover, a higher entropy value (≥0.8 indicates more than 90% classification accuracy) indicated a more accurate model classification. LMR and BLRT were used to compare whether the difference between the *k* and *k* − 1 categories was significant, and a smaller *p*-value indicated a better model *k* category. A multivariate logistic regression model was used to test the relationships between different dietary trajectories and BMI changes. Statistical analysis was performed by using Mplus 8.3 and SPSS software version 26.0.

## 3 Results

### 3.1 General information of the research subjects

A total of 2,465 elderly people aged 65 years and older were included in this study at baseline, and a total of 1,349 respondents with complete data were followed after 7 years. There was no statistically significant difference between the baseline characteristics of the older adults who completed follow-up and the baseline characteristics of the overall study subjects, as shown in Table 1. Among the 1,349 elderly people, the average age was 81.21 (SD = 8.9) years, 45.96% were female, 42.40% received formal education, 83.99% were married, 56.34% were rural, 67.01% were low- and middle-income people, 74.65% were living with their families, 29.95% were smokers, 42.25% were drinkers, 32.39% were less active, and 20.31% of older people with 2 or more chronic diseases had tooth loss. General information on the study subjects is shown in Table 1.

**Table 1:** Baseline characteristics of the study subjects and those who completed follow-up.

Baseline characteristics	Baseline respondents (*N* = 2,465), *n*/(%)	Follow-up respondents (*N* = 1,349), *n*/(%)	*χ*^2^^a^	*p*
Age			1.172	0.279
65–79	1763 (71.52)	987 (73.17)		
≥80	702 (28.48)	362 (26.83)		
Sex			3.628	0.057
Male	1,411 (57.24)	729 (54.04)		
Female	1,054 (42.76)	620 (45.96)		
Residence			1.426	0.232
Rural	1,438 (58.34)	760 (56.34)		
Town	1,027 (41.66)	589 (43.66)		
Marital status			0.064	0.801
With spouse	2078 (84.30)	1,133 (83.99)		
No spouse	387 (15.70)	216 (16.01)		
Education			0.001	0.985
Formal	1,046 (42.43)	572 (42.40)		
Nonformal	1,419 (57.57)	777 (57.60)		
Economic			0.280	0.597
Low and middle	1,631 (66.17)	904 (67.01)		
High	834 (33.83)	445 (32.99)		
Smoke			2.784	0.095
Yes	803 (32.58)	404 (29.95)		
No	1,662 (67.42)	945 (70.05)		
Drink			0.001	0.989
Yes	1,041 (42.23)	570 (42.25)		
No	1,424 (57.77)	779 (57.75)		
Living together			1.421	0.492
Living with family	1831 (74.28)	1,007 (74.65)		
Nursing home	52 (2.11)	21 (1.56)		
Living alone	582 (23.61)	321 (23.79)		
Exercise			0.004	0.949
Yes	801 (32.49)	437 (32.39)		
No	1,664 (67.51)	912 (67.61)		
Chronic disease			0.094	0.760
Yes	511 (20.73)	274 (20.31)		
No	1954 (79.27)	1,075 (79.69)		
Tooth loss			2.471	0.116
Yes	1,543 (62.60)	879 (65.16)		
No	922 (37.40)	470 (34.84)		

a The differences in these characteristics between the analyzed sample and baseline sample were evaluated using chi-square tests.

### 3.2 Analysis of the development trajectory of the diet of elderly people

The LGMM results showed that the AIC, BIC and aBIC values were smaller and that the entropy values changed accordingly, which indicated that there was heterogeneity in the longitudinal data population. In principle, the 5-category model should be retained; however, only 0.1% of patients with categories were found in the 5-category model, 2 potential categories were found to be very similar, and the distinction was not obvious. When considering the simplicity of the model, the 4-category model was ultimately retained, as shown in Table 2.

**Table 2:** LGMM fitting information of the dietary trajectories of elderly people in the community.

Category	AIC	BIC	aBIC	Entropy	LMR	BLRT	Category probability
C1	6081.782	6063.113	6031.339				1
C2	5970.901	6022.972	5911.206	0.703	<0.001	<0.001	0.429/0.571
C3	5964.215	6031.907	5900.612	0.931	0.0013	<0.001	0.471/0.106/0.423
C4	5564.847	5648.161	5597.336	0.968	<0.001	<0.001	0.202/0.288/0.378/0.132
C5	4918.172	5017.107	4956.252	0.971	<0.001	<0.001	0.001/0.682/0.057/0.001/0.259

### 3.3 Nomenclature and characteristics of the categories of dietary development trajectories of elderly people

**Figure 1:**
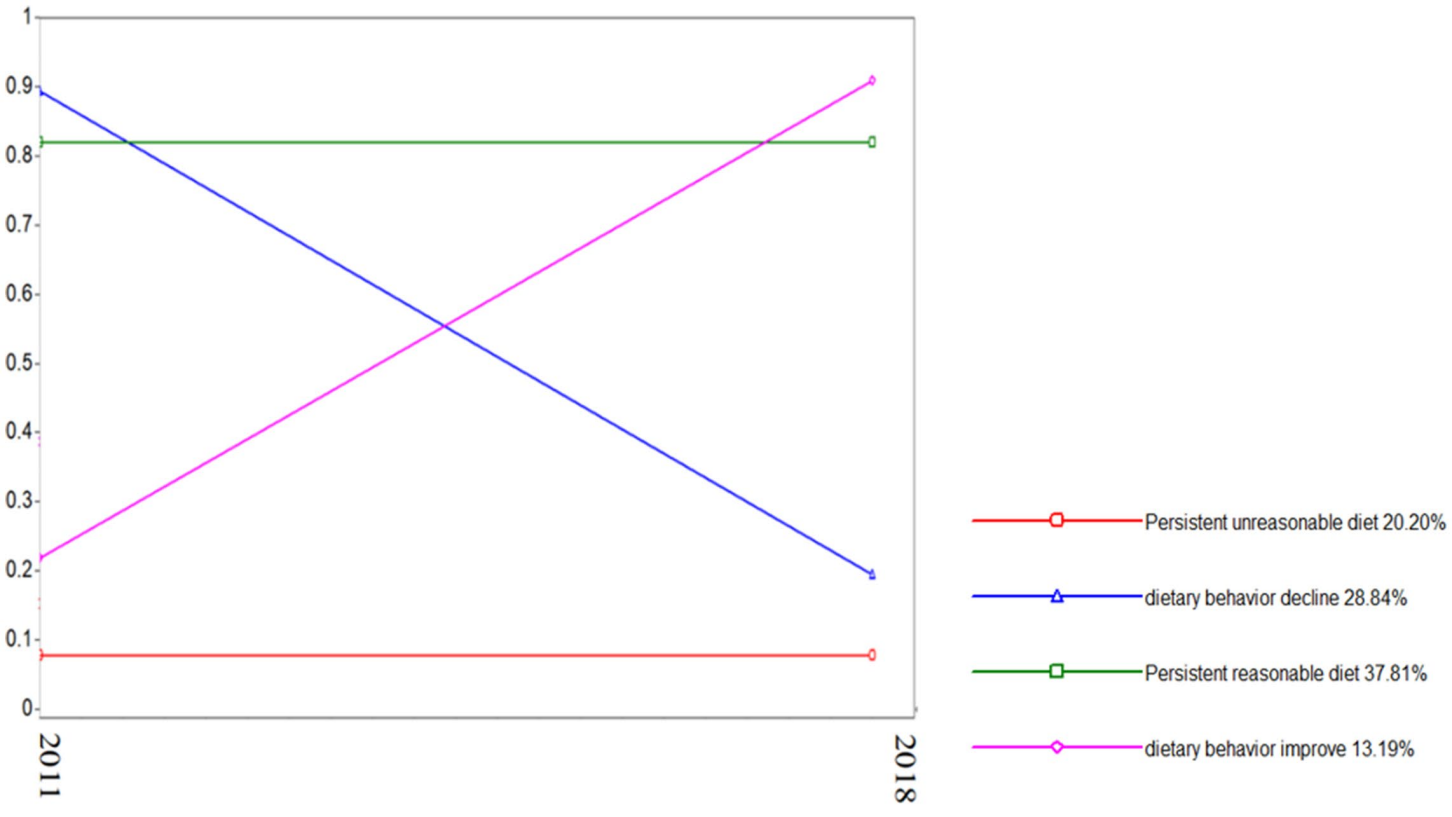
Reasonable diet trajectory for elderly people in the community.

A total of 1,349 elderly patients were included in this study; these patients could be divided into four groups, as shown in Figure 1. The first category was termed the “persistent unreasonable diet group,” and the dietary evaluation of the elderly individuals in this category was unreasonable at baseline and follow-up, with a total of 272 people (accounting for 20.20%). The second category was termed the “Dietary Behavior Decline Group,” and 389 elderly people in this category were evaluated as reasonable at baseline and unreasonable after 7 years of follow-up, accounting for 28.84%. The third category was the “continuous reasonable diet group,” and the dietary behavior of the elderly individuals in this group was reasonable at baseline and follow-up, with a total of 510 people (accounting for 37.81%). The fourth category was the “dietary behavior improvement group,” and there were 178 elderly people in this category, accounting for 13.19%; the dietary behavior of these individuals was unreasonable at baseline but improved after follow-up.

Univariate analysis of demographic and clinical characteristics among the four categories of dietary development trajectories of the elderly showed that there were significant differences in age, sex, place of residence, education level, marital status, economic status, alcohol consumption, cohabitants, physical exercise, and comorbidities among the different categories (*p* < 0.05). There was no significant difference in smoking status among the four categories, as shown in Table 2. The four categories determined via LGMM analysis were used as dependent variables; moreover, the meaningful variables of the univariate analysis were used as independent variables, and multivariate logistic regression analysis was performed with “continuous reasonable diet group” as the reference category, as shown in Table 3. The results showed that males (OR = 1.614, 95% CI = 1.167–2.233, *p* = 0.004), rural residents (OR = 0.498, 95% CI = 0.361–0.685, *p* < 0.001), people with no spouses (OR = 0.344, 95% CI = 0.195–0.609, *p* = 0.011), people with no formal education (OR = 1.480, 95% CI = 1.072–2.045, *p* = 0.017), people who were not wealthy (OR = 2.368, 95% CI = 1.496–3.749, *p* < 0.001), people who lived alone (OR = 2.652, 95% CI = 1.676–4.195, *p* < 0.001), and people who experienced tooth loss (OR = 1.036, 95% CI = 0.440–2.438, *p* = 0.036) were more likely to be in the “continuous unreasonable diet group” (*p* < 0.05) (see Table 4).

**Table 3:** Relationships between dietary trajectories and sociodemographics of elderly people in the community.

Variable^a^	Total (*n* = 1,349)	Persistent unreasonable diet (*n* = 272)	Dietary behavior decline (*n* = 389)	Continuous reasonable diet (*n* = 510)	Dietary behavior improve (*n* = 178)	*χ* ^2^	*p*
Age						16.922	0.001
65–79	796 (59.01)	160 (58.82)	231 (59.38)	277 (54.31)	128 (71.91)		
≥80	553 (40.99)	112 (41.18)	158 (40.62)	233 (45.69)	50 (28.09)		
Sex						24.008	<0.001
Male	729 (54.04)	122 (44.85)	226 (58.10)	302 (59.22)	79 (44.38)		
Female	620 (45.96)	150 (55.15)	163 (41.90)	208 (40.78)	99 (55.62)		
Residence						16.898	0.001
Rural	739 (54.78)	179 (65.81)	209 (53.73)	275 (53.92)	116 (65.17)		
Town	610 (45.22)	93 (34.19)	180 (46.27)	235 (46.08)	62 (34.83)		
Marital status							
With spouse	1,061 (81.62)	229 (84.19)	304 (78.15)	411 (80.59)	117 (88.20)	23.867	<0.001
No spouse	288 (18.38)	43 (15.81)	85 (21.85)	99 (19.41)	61 (11.80)		
Education						11.046	0.011
Formal	572 (42.40)	94 (34.56)	173 (44.47)	235 (46.08)	70 (39.33)		
Nonformal	777 (57.60)	178 (65.44)	216 (55.53)	275 (53.92)	108 (60.67)		
Economic status						32.056	<0.001
Low and middle	1,104 (81.84)	244 (89.71)	302 (77.63)	396 (77.65)	162 (91.01)		
High	245 (18.16)	28 (10.29)	87 (22.37)	114 (22.35)	16 (8.99)		
Smoke or not						5.532	0.137
Yes	193 (14.31)	46 (16.91)	53 (13.62)	62 (12.16)	32 (17.98)		
No	1,156 (85.69)	226 (83.09)	336 (86.38)	448 (87.84)	146 (82.02)		
Drinking status						12.578	0.006
Yes	233 (17.27)	52 (19.12)	85 (21.85)	67 (13.14)	29 (16.29)		
No	1,116 (82.73)	220 (80.88)	304 (78.15)	443 (86.86)	149 (83.71)		
living together						43.439	<0.001
Living with family	947 (70.20)	170 (62.50)	265 (68.12)	361 (70.78)	151 (84.83)		
Nursing home	30 (2.22)	3 (1.10)	5 (1.29)	14 (2.75)	8 (4.49)		
Living alone	372 (27.58)	99 (36.40)	119 (30.59)	135 (26.47)	19 (10.68)		
Exercise						16.041	<0.001
Yes	543 (38.25)	86 (31.62)	147 (37.79)	229 (44.90)	81 (45.51)		
No	806 (61.75)	186 (68.38)	242 (62.21)	281 (55.10)	97 (54.49)		
Chronic disease						98.948	<0.001
Yes	398 (29.50)	79 (29.04)	90 (23.14)	121 (23.73)	108 (60.67)		
No	951 (70.50)	193 (70.96)	299 (76.86)	389 (76.27)	70 (39.33)		
Tooth loss						116.032	<0.001
Yes	902 (66.86)	218 (80.15)	317 (81.49)	271 (53.14)	96 (53.93)		
No	447 (33.14)	54 (19.85)	72 (18.51)	239 (46.86)	82 (46.07)		

a The number of sociodemographic variables in the population after 7 years of follow-up, and the number of variables at baseline and follow-up is shown in Table 1.

**Table 4:** Factors influencing the dietary trajectories of elderly people in the community.

Variable	Persistent unreasonable diet			Dietary behavior decline			Dietary behavior improvement		
	OR 值	95% CI	*p*-值	OR 值	95% CI	*p*-值	OR 值	95% CI	*p*-值
*Age*									
65–79	Ref			Ref			Ref		
≥80	1.047	0.762–1.438	0.777	1.544	1.065–2.238	0.022	1.144	0.794–1.648	0.471
*Sex*									
Female	Ref			Ref			Ref		
Male	1.614	1.167–2.233	0.004	1.113	0.837–1.479	0.462	0.364	0.220–0.601	0.552
*Residence*									
Town	Ref			Ref			Ref		
Rural	0.498	0.361–0.685	<0.001	0.788	0.600–1.034	0.085	0.712	0.417–1.214	0.212
*Marital status*									
With spouse	Ref			Ref			Ref		
No spouse	0.344	0.195–0.609	0.011	0.277	0.141–0.546	<0.001	0.997	0.579–1.715	0.990
*Education*									
Formal	Ref			Ref			Ref		
Nonformal	1.480	1.072–2.045	0.017	1.016	0.772–1.336	0.910	1.175	0.815–1.694	0.387
*Economic*									
High	Ref			Ref			Ref		
Low and middle	2.368	1.496–3.749	<0.001	0.983	0.711–1.361	0.020	0.606	0.369–0.995	0.058
*Living together*									
Living with family	Ref			Ref			Ref		
Nursing home	0.701	0.186–2.635	0.599	0.366	0.046–2.933	0.344	2.052	1.221–3.449	0.097
Living alone	2.652	1.676–4.195	<0.001	2.446	1.475–4.057	0.051	1.192	0.739–1.923	0.471
*Drinking status*									
No	Ref			Ref			Ref		
Yes	2.071	1.359–3.156	0.091	1.944	1.354–2.790	<0.001	1.654	1.007–2.718	0.082
*Exercise*									
No	Ref			Ref			Ref		
Yes	1.620	1.141–2.302	0.007	2.616	1.487–4.602	0.061	1.584	1.141–2.200	0.056
*Chronic disease*									
No	Ref			Ref			Ref		
Yes	1.137	0.784–1.647	0.059	1.296	0.980–1.713	0.069	0.740	0.465–1.179	0.025
*Tooth loss*									
No	Ref			Ref			Ref		
Yes	1.036	0.440– 2.438	0.036	1.084	0.665–1.768	<0.001	0.648	0.418–1.002	0.078

### 3.4 Relationship between dietary trajectory category and BMI change in elderly people

**Figure 2:**
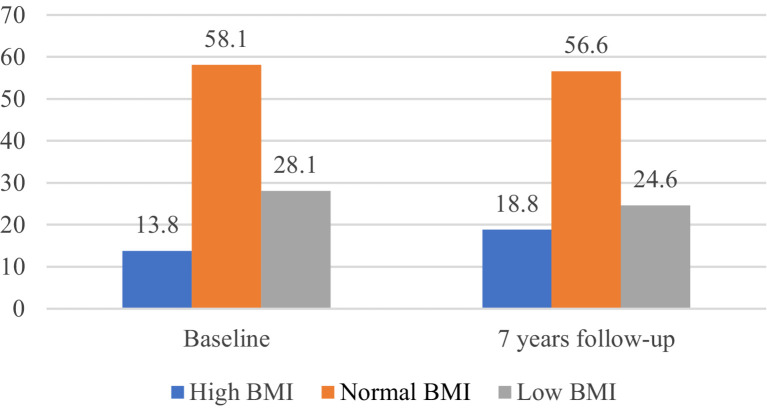
BMI status of elderly people in the community at baseline and at the 7-year follow-up.

At baseline, the elderly individuals were divided into thin, normal, and obese groups according to the appropriate BMI range proposed in the Dietary Guidelines for the Elderly (5). The ratio of BMI at baseline to that at follow-up is shown in Figure 2. In this study, nine BMI change patterns were constructed according to the BMI at baseline and follow-up, including the “thin stable group” (156 patients), “normal stable group” (513 patients), “thin-normal group” (180 patients), “thin-obese group” (43 patients), “normal-thin group” (161 patients), “normal-obesity group” (109 patients), “obesity-stable group” (101 patients), “obese-thin group” (15 patients), and “obese-normal group” (71 patients).

Multivariate logistic regression was used to explore the relationship between dietary trajectory categories and BMI changes in elderly individuals, and the results showed that when the covariates were not adjusted, the BMIs of the elderly individuals in the “continuous reasonable diet group” and the “dietary behavior improvement group” tended to be normal compared with those in the “continuous unreasonable diet group” (“continuous reasonable diet group,” “normal and stable group”: OR = 1.071, 95% CI = 0.626–1.832, *p* = 0.003; “obesity-normal group”: OR = 1.029, 95% CI = 0.437–2.426, *p* = 0.041). The inclusion criteria for the “dietary behavior improvement group” were also determined (“normal and stable group”: OR = 0.796, 95% CI = 0.418–1.514, *p* = 0.026; “thin-normal” group: OR = 0.421, 95% CI = 0.194–0.912, *p* = 0.028; and “obesity-normal group”: OR = 1.136, 95% CI = 0.422–3.063, *p* = 0.001). The BMI of the elderly individuals in the “dietary behavior decline group” tended to be lean (“normal-lean group”: OR = 2.567, 95% CI = 1.127–5.849, *p* = 0.006). After adjusting for age, sex, place of residence, education level, marital status, economic status, smoking status, alcohol consumption status, living with people, exercise status, tooth loss status, and comorbidities, improvements in continuous reasonable diet and dietary behavior were significantly correlated with the development of a normal BMI (“continuous reasonable diet group,” “normal stable group”: OR = 1.907, 95% CI = 1.356–2.682, *p* < 0.001; “obese-normal group”: OR = 0.859, 95% CI = 0.350–1.458, *p* = 0.039) (“dietary behavior improvement group,” “thin-normal group”: OR = 0.412, 95% CI = 0.188–0.902, *p* = 0.027; “obese-normal group”: OR = 1.241, 95% CI = 0.448–3.437, *p* < 0.001). A decrease in dietary behavior was significantly associated with a change from a normal BMI to a lean BMI (“normal-lean group”: OR = 1.117, 95% CI = 1.034–2.402, *p* = 0.001), as shown in Table 5.

**Table 5:** The relationship between the trajectory of reasonable diet behavior and changes in BMI among elderly people in the community.

Group	Model 1			Model 2			Model 3		
	OR	95% CI	*p*	OR	95% CI	*p*	OR	95% CI	*p*
*Continued reasonable diet*									
Lean stable	Ref			Ref			Ref		
Normal stable	1.071	0.626–1.832	0.003	0.937	0.539–1.630	0.001	1.907	1.356–2.682	<0.001
Thin-normal	0.692	0.379–1.266	0.052	0.753	0.405–1.401	0.074	1.824	1.138–2.923	0.092
Thin-obese	0.535	0.230–1.240	0.145	0.487	0.202–1.175	0.220	0.509	0.209–1.240	0.137
Normal-lean	0.836	0.445–1.570	0.577	0.775	0.404–1.485	0.442	0.754	0.390–1.458	0.402
Normal-obese	0.623	0.308–1.259	0.187	0.576	0.278–1.191	0.136	0.586	0.281–1.224	0.155
Stable obese	0.879	0.435–1.776	0.719	0.768	0.368–1.603	0.482	0.736	0.349–1.554	0.421
Obesity-lean	1.415	0.352–5.684	0.055	2.216	0.503–8.976	0.092	2.614	0.605–11.295	0.150
Obesity-normal	1.029	0.437–2.426	0.041	0.877	0.360–2.133	0.049	0.859	0.350–1.458	0.039
*Dietary behavior decline*									
Lean stable	Ref			Ref			Ref		
Normal stable	0.801	0.466–1.376	0.422	0.763	0.438–1.328	0.339	0.567	0.431–1.324	0.328
Thin-normal	0.836	0.536–1.304	0.430	0.573	0.296–1.112	0.100	0.635	0.307–1.312	0.220
Thin-obese	0.123	0.037–0.410	0.051	0.638	0.312–1.307	0.220	0.551	0.283–1.076	0.081
Normal-lean	2.567	1.127–5.849	0.006	0.114	0.033–0.389	0.001	1.117	1.034–2.402	0.001
Normal-obese	0.595	0.311–1.138	0.067	0.489	0.258–0.925	0.028	0.464	0.244–0.885	0.120
Stable obese	0.630	0.312–1.269	0.055	0.560	0.261–1.200	0.074	0.534	0.247–1.156	0.112
Obesity-lean	0.309	0.048–1.965	0.213	0.433	0.066–2.816	0.381	0.509	0.077–3.381	0.485
Obesity-normal	1.010	0.429–2.379	0.982	0.923	0.382–2.230	0.859	0.864	0.355–2.104	0.748
*Dietary behavior improve*									
Lean stable	Ref			Ref			Ref		
Normal stable	0.796	0.418–1.514	0.026	0.788	0.412–1.508	0.031	0.835	0.434–1.605	0.058
Thin-normal	0.421	0.194–0.912	0.028	0.410	0.189–0.893	0.025	0.412	0.188–0.902	0.027
Thin-obese	0.486	0.169–1.400	0.181	0.472	0.161–1.385	0.172	0.485	0.164–1.430	0.189
Normal-lean	0.565	0.256–1.247	0.018	0.560	0.253–1.240	0.053	0.573	0.257–1.276	0.173
Normal-obese	0.708	0.308–1.629	0.417	0.696	0.300–1.612	0.398	0.746	0.320–1.741	0.498
Stable obese	0.521	0.209–1.301	0.162	0.515	0.203–1.308	0.163	0.567	0.221–1.452	0.237
Obesity-lean	0.347	0.034–3.574	0.374	0.334	0.032–3.478	0.359	0.386	0.037–4.072	0.429
Obesity-normal	1.136	0.422–3.063	0.001	1.180	0.430–3.234	<0.001	1.241	0.448–3.437	<0.001

Model 1, not adjusted; Model 2, adjusted for age and sex; Model 3, corrected for sex, age, place of residence, education, smoking status, alcohol consumption status, marital status, economic status, exercise status, tooth loss status, and history of comorbidities.

## 4 Discussion

### 4.1 Categorical characteristics of dietary development trajectories in the elderly

In this study, the LGMM model was used to assess the change trajectory and factors influencing the diet of elderly people in the community. The model fit a total of four dietary trajectories: the continuous reasonable diet group, the dietary behavior decline group, the continuous unreasonable diet group, and the dietary behavior improvement group.

The “dietary behavior improvement group” accounted for the lowest proportion, which was 13.19%, and the baseline dietary evaluation of the elderly individuals in this category was unreasonable; however, their dietary behavior improved at follow-up. The results of regression analysis showed that this group was dominated by older people with comorbidities; specifically, 108 elderly people in this category had comorbidities, of which 81 (74.1%) had diabetes and hypertension at the same time. Dietary control is the main nonpharmacological treatment for diabetes and hypertension. With the promotion of the Healthy China Initiative and the promotion of dietary guidelines for residents, elderly people have begun to understand the importance of a reasonable diet; moreover, when they learn that they have diabetes or high blood pressure, they will consciously improve their dietary behavior. In this category, 129 (72.5%) of the elderly individuals reduced their intake of staple foods; 134 (75.3%) of the elderly individuals reduced their intake of sugar, salt and pickled products; and 152 (85.4%) of the elderly individuals increased their intake of vegetables, fruits, fish, eggs and other foods. For this group of elderly people, it is necessary to encourage them to continue to maintain the enthusiasm and continuity of a reasonable diet (22, 23).

The proportion of the “dietary behavior decline group” was 28.84%, and the baseline dietary evaluation of the elderly individuals in this category was reasonable; however, the dietary evaluation was unreasonable at follow-up. The results of regression analysis showed that this group of people was mainly unmarried, low- and middle-income, alcoholic, and elderly with tooth loss. With increasing age, the physical and mental functions of elderly people, such as the decline of smell, taste and other sensations, absorption and digestion functions, coupled with the loss of teeth, as well as modulation of some food choices, decline to varying degrees. This results in a gradual decrease in dietary intake and types, especially the insufficient intake of fruits, dairy products, nuts and soy products. A total of 274 (70.4%) elderly people in this category reduced their intake of fruits, dairy products, nuts and soy products. Marriage has a considerable impact on the diet of older adults, which is consistent with the findings of most studies (24). Among the unmarried elderly individuals in this category, 82 (82.83%) were widowed. Zhao and Li (25) used 6-phase CLHLS data to analyze the impact of widowhood on multiple health outcomes and health behaviors and showed that widowhood can lead to changes in the original living status of elderly people, as well as reduce the available psychological and social support resources, increase the risk of depression and cognitive decline in elderly people, and lead to decreased appetite. Older adults who live alone after widowhood may reduce their intake of nutritious food due to a lack of financial support (26). Furthermore, widows are more likely to engage in health-risk behaviors such as alcohol consumption, which is due to the need to relieve widowhood stress and the lack of spousal supervision (27).

The “persistent unreasonable diet group” accounted for 20.16% of the sample, and the dietary evaluation of the elderly individuals in this category was unreasonable at baseline and at follow-up. The results of regression analysis showed that this group of people was mainly male, lived in rural areas, did not have formal education, had low incomes, lived alone, and had tooth loss. The dietary habits of older men were worse than those of older women, which was consistent with the findings of most studies. Feraco et al. (28) investigated sex differences in the eating habits of 2,388 adults and showed that women had better eating habits than men. In a study of healthy eating among 262 respondents conducted by Razaz et al. (29), women were more likely to adopt a healthy diet than men were (OR = 1.85, 95% CI = 1.02–3.35). The analysis of the sex differences in this study may be due to the following reasons. First, differences in dietary choices between men and women. In this category, men preferred spicy and salty foods, and the number of people who regularly consumed lard (76 cases, 27.94%) was significantly greater than that of women (28 cases, 10.29%). Only 16.91% of men regularly consumed soy products, fruits, and vegetables, which may be related to evolutionary dietary roles and current social norms (28). Second, men have a greater rate of social participation than women and often rely on out-of-home gatherings to build or strengthen their social support networks, which also indirectly leads to poorer eating control behaviors. Older people living in rural areas, with no formal education and with low incomes, have less access to information on healthy diets and lack knowledge about nutrition and health, although the state is actively publicizing the impact of a reasonable diet on health. Due to the limitations of the living environment, some elderly people may not be aware of the important role of nutrition in health and are unwilling to accept nutritional knowledge and change their dietary habits for reasonable nutrition. Most of the elderly people are mainly engaged in farm work, with greater energy consumption and increased intake of staple foods, oil and salt but less intake of milk, eggs and fruits. Second, due to economic constraints, low-income elderly people will reduce their demand for dairy products, meat, eggs, fish and nuts, thus resulting in a greater risk of malnutrition. Older people who live alone may have irregular diets and lower intake of important nutrients; thus, they are more likely to suffer from malnutrition due to lack of care from family members or financial constraints.

### 4.2 Relationship between dietary trajectory category and BMI change in older adults

The results of this study demonstrated that a “continuous reasonable diet group” and “dietary behavior improvement group” were significantly associated with the development of obesity in individuals with a normal BMI. For obese older adults, a reasonable diet has a positive effect on maintaining a normal BMI, which is consistent with the results of most studies. Acharya et al. (30) conducted a one-year dietary intervention in 80 obese middle-aged and elderly people and showed that a low-oil, low-fat, and low-calorie diet could reduce body weight by 2.9%, which was meaningful for maintaining normal body weight (OR = −2.5, 95% CI = −6.289 to 1.289). Lu and Zhang (31) conducted a 4-month nondrug nutritional intervention on 68 overweight and obese elderly people and compared body weight and other indicators before and after the intervention. After 4 months of nonpharmacological nutritional intervention, the weight of the elderly individuals decreased to varying degrees; moreover, the BMI also improved, and most of the elderly individuals changed from being obese to having a normal weight. According to the Weight Loss Action of Healthy Weight Management in China, a reasonable diet is the basis for scientific weight loss (32). The National Health Commission also proposed that the most important way to scientifically lose weight is to “keep your mouth shut,” 80% of which rely on a reasonable diet; furthermore, exercise is only used as an auxiliary function. The reasonable diet mentioned in the “Dietary Guidelines for the Elderly” should include cereals and potatoes, vegetables and fruits, livestock, poultry, fish, eggs and milk and legumes as a daily diet; moreover, individuals should reduce the intake of oil, salt and sugar, as well as appropriately increase the intake of dairy products. With age, older people eat less and eat cereals and potatoes as staple foods, which reduces the intake of other high-calorie foods, such as high oil and sugar. High-protein diets such as livestock, poultry, fish, and eggs mainly use the satiety effect of protein to reduce energy intake and body fat mass and improve the metabolic status of elderly people (33). Milk and its products are the best sources of dietary calcium, thus providing a variety of essential nutrients; moreover, an appropriate daily increase in dairy products in elderly people can help to reduce weight (32). Vegetables, fruits and soy products can help to increase the satiety of elderly people, as well as reduce the intake of high-calorie, high-fat foods that are rich in dietary fiber and slow the increase in blood sugar after meals. This scenario ultimately plays an important role in weight control. This study did not demonstrate an effect of a reasonable diet on the development of a lean to normal weight, which may be due to the fact that the change in BMI trajectory in elderly individuals is not easy to considerably change in a short period of time; moreover, in elderly individuals, a thin weight at baseline, tooth loss, a decrease in chewing function and other factors lead to a decrease in food intake, insufficient nutrient intake, and difficulty in returning to normal weight in a short period of time due to physical fitness and other reasons. In the future, other studies will investigate the dietary trajectory of thin older adults and explore its relationship with BMI.

### 4.3 Future recommendations for older populations with different dietary trajectory categories

This study explores the trajectory of reasonable diet among the elderly in the community in China, and finds the characteristics of different trajectory categories, which should not only be observed from the old age, but should be examined throughout the life course of the individual (34). In the context of promoting healthy ageing in the world, we must adopt active and effective personalized strategies to improve the existing problem of unreasonable diets of the elderly with different characteristics.

The basic health rights and interests of elderly people in low social classes should be protected; moreover, elderly people in rural areas who are old, have a low level of education, and have a poor economic status should be considered as the bottom group of China’s basic social security system. Therefore, the health service system for elderly people should be improved, and the quality of health services for elderly people should be expanded in terms of content, region, and coverage of infrastructure. These interventions should include the prevention and treatment of overnutrition or malnutrition, as well as local medical and health service policies, the government should strengthen food security for vulnerable groups, and the “Health and Nutrition Report for the Elderly” proposes that food stamps can be used as a meal assistance program (2), which can provide more choices to recipients, increase food spending for low-income people, release part of their purchasing power, ensure their healthy diet and improve their nutritional level.

At present, most parts of the country have set up canteens for elderly people. The purpose of these canteens is not only to provide rich and healthy nutritious meals for elderly people to meet their nutritional needs but also to provide a place for them to exchange ideas and gather together (35). However, the development status of the elderly canteen is not optimistic. This may be related to food not meeting the habits or needs of different older people. Thus, government departments should actively optimize the structure of the canteen for elderly people and provide personalized meal services for elderly people of different ages according to their needs and dining tastes, especially elderly people who have lost their teeth and who should be provided with soft and fine food, nuts or fruits that can be ground into powder or juiced for consumption. Canteens for elderly people should provide preferential policies for these individuals. For elderly people who live far from the canteen, the community can provide them with free meal delivery services to avoid the intake of food and drink due to inconvenient activities. Moreover, in the case of reduced dietary intake, according to the guidance of professionals, appropriate nutrients can be selected to meet the needs of elderly people for trace elements.

The community has gradually established and standardized the health records of elderly people with chronic diseases in the community and has provided elderly people with an inclusive package of contracted family doctor services; in particular, elderly people with chronic disease or hypertension are provided with free follow-up visits four times a year, which include regular blood sugar and blood pressure measurements and disease-related health education and health literacy services (36). Due to the fact that the participation of family doctor contract services is voluntary, the participation rate of elderly people may be low for various reasons; therefore, community workers should actively publicize and popularize family doctor contract services to elderly people so that elderly people can actively participate and improve their ability to perceive the disease to better manage their diet.

The communities should actively respond to the national campaign policy, carry out community mobilization activities in the form of posters or banners, encourage the elderly in the community to actively participate in reasonable dietary health actions, make them aware of their own health responsibilities, change unhealthy health-related behaviors and lifestyles, enhance the awareness of reasonable diet among the elderly in the community, strengthen dietary and nutrition education, and provide various opportunities for them to regularly participate in health education activities, learn and master dietary health-related knowledge and skills in the participation. Choose to vigorously publicize in a form that is easily accepted by the elderly, such as television and broadcasting, to provide the elderly with more specific, practical and easy-to-operate nutritional information, as well as free and authoritative dietary guidance and consultation.

### 4.4 Strengths and weaknesses

This study discussed the dietary development trajectory of the elderly in the community in China, analyzed the influencing factors of different dietary trajectories of the elderly. Diet is significantly correlated with BMI, and reasonable dietary behavior is an important measure to prevent and improve overweight or obesity in the elderly. This study provides a reference for the elderly in the community to formulate scientific personalized interventions and cultivate healthy living habits. The advantages of this study are as follows. First, the participants of this study were elderly individuals in the CLHLS database, and the respondents originated from multiple regions; therefore, the study population is more representative. Second, this study explored the dietary trajectory of elderly individuals for the first time; additionally, based on the Healthy China Action (2019–2030), it was shown that a reasonable diet is the most effective way to maintain normal weight. This study verified that the correlation between different dietary trajectories and BMI of the elderly people in the community, thus providing insights for elderly individuals to formulate scientific and reasonable dietary interventions. There are also some shortcomings in this study. First, the self-reported information that was collected by using the Food Frequency Questionnaire is prone to recall bias. Second, the questionnaire only collected information about the frequency of food intake, rather than the specific amount of food intake, especially the lack of food intake such as oil, salt, and meat, which led to the failure to account for the problem of excessive or too little caloric intake in the unreasonable diet when classifying whether it is a reasonable dietary behavior, which may bias the results. Thirdly, the BMI range of the general elderly (65–79 years old) in this study is defined as 20 kg/m^2^–26.9 kg/m^2^, but some guidelines or studies have found that the optimal BMI range for the elderly is 22 kg/m^2^–26.9 kg/m^2^, and it is believed that the lower BMI range of the elderly should be increased, which may be due to the influence of geography or cultural customs, in the future, this study will continue to explore the optimal BMI range for the elderly. At present, there are very few studies on sex or age differences in the dietary nutrition trajectories of elderly people in the community; moreover, due to differences in physical constitution or body structure, the manner of how men and women differ in dietary nutrition trajectories should be the focus of our future research. Past studies have typically explored the effects of dietary management on obese older adults. Currently, the incidence of malnutrition in elderly individuals is gradually increasing, and malnutrition can also cause a variety of chronic diseases and even increase the risk of death; therefore, malnourished elderly individuals are still the key group to which we need to pay attention, and the trajectory of weight change in malnourished elderly individuals needs to be further explored.

## 5 Conclusion

Based on the framework of the Healthy China Action (2019–2030), this study discussed the development trajectory of four community diets for the elderly population, indicating the heterogeneity of the elderly population’s dietary behavior, thus suggesting that we should formulate or adopt different intervention strategies according to different categories of elderly populations. Regularly carry out nutritional screening and assessment, according to the nutritional status of elderly people, combined with their daily diet and living habits, to provide appropriate and targeted nutritional knowledge and daily dietary advice. This study also explored the relationship between different trajectories and changes in BMI in elderly people, and the results confirmed that the correlation between different dietary trajectories and BMI of the elderly people in the community, which will help to provide an appropriate entry point for the formulation of public health policies. However, this study has not yet investigated the effect of a reasonable diet on low-BMI elderly individuals, and we will further explore the effect of a reasonable diet on the weight change trajectory of malnourished older adults in the future.

## Data availability statement

The raw data supporting the conclusions of this article will be made available by the authors, without undue reservation.

## Ethics statement

The data used in this study was obtained from the CLHLS, which was managed by the Peking University Center for Healthy Aging and Development Studies. All procedures were carried out in accordance with relevant guidelines and regulations (Declaration of Helsinki). All individuals provided written informed consent before participating in the study. Written informed consent was obtained from the individual(s) for the publication of any potentially identifiable images or data included in this article.

## Author contributions

ML: Conceptualization, Data curation, Formal analysis, Funding acquisition, Investigation, Methodology, Project administration, Resources, Software, Supervision, Validation, Visualization, Writing – original draft, Writing – review & editing. YC: Data curation, Writing – review & editing. WG: Formal analysis, Writing – review & editing. SZ: Data curation, Writing – review & editing. MZ: Methodology, Writing – review & editing. XM: Software, Writing – review & editing. XJ: Supervision, Writing – review & editing. YL: Data curation, Writing – review & editing. LZ: Formal analysis, Funding acquisition, Validation, Writing – review & editing.

## References

[1] National Bureau of Statistics of the People’s Republic of China. National data-2022 annual data National Bureau of Statistics of the People’s Republic of China (2022) Available at: http://www.stats.gov.cn/sj/ndsj/.

[2] Research on Nutrition and Health of the Elderly in China-China Development Research Foundation. Report on nutrition and health of the elderly in China China Development Research Foundation (2015) Available at: http://www.cdrf.org.cn/zglnr/index.htm.

[3] Zhang J. Nutrition of the elderly in China: a review and prospect in the past ten years. Health Res. (2022) 51:692–5. doi: 10.19813/j.cnki.weishengyanjiu.2022.05.002

[4] Shimizu I Yoshida Y Minamino T. The significance of lipid deficiency in cardiometabolic diseases. Phys Fitness Sci. (2021) 70:71–1. doi: 10.7600/jspfsm.70.71

[5] Zhang C. Chinese nutrition society released dietary guidelines for Chinese residents. Food Saf Guide. (2022) 14:4. doi: 10.16043/j.cnki.cfs.2022.14.059

[6] Han YH. Study on the prognostic value of body mass index in elderly patients with comorbidities Qingdao University. doi: 10.27262/d.cnki.gqdau.2021.000142

[7] Lv Y Zhang Y Li X Gao X Ren Y Deng L. Body mass index, waist circumference, and mortality in subjects older than 80 years: a Mendelian randomization study. Eur Heart J. (2024) 45:2145–54. doi: 10.1093/eurheartj/ehae206 38626306 PMC11212828

[8] Wei C Hua J. 2016 Chinese overweight/obesity medical nutrition therapy expert consensus interpretation. Chin J Pract Intern Med. (2017) 37:430–3. doi: 10.19538/j.nk2017050117

[9] Zheng H Echave P Mehta N Myrskylä M. Life-long body mass index trajectories and mortality in two generations. Ann Epidemiol. (2021) 56:18–25. doi: 10.1016/j.annepidem.2021.01.003, PMID: 33493649 PMC8009819

[10] Wang ZQ Zhang M Zhao YF Yang J Zhao WH. Incidence and 20-year trend of underweight malnutrition in the elderly population of China. Disease Surveill. (2014) 29:477–80.

[11] Seko Y Kato T Morimoto T Yaku H Inuzuka Y Tamaki Y. Association between body mass index and prognosis of patients hospitalized with heart failure. Sci Rep. (2020) 10:16663. doi: 10.1038/s41598-020-73640-w 33028856 PMC7542148

[12] Park HJ Cho JH Kim HJ Park JY Lee HS Byun MK. The effect of low body mass index on the development of chronic obstructive pulmonary disease and mortality. J Intern Med. (2019) 286:573–82. doi: 10.1111/joim.12949 31215064

[13] Chinese Nutrition Society. Appropriate range of body mass index and body weight management guidelines for Chinese oldest old (T/CNSS 021-2023). Chin J Epidemiol. (2023) 44:1335–7. doi: 10.3760/cma.j.cn112338-20230804-00055 37743262

[14] Zeng Y. Towards deeper research and better policy for healthy aging—using the unique data of Chinese longitudinal healthy longevity survey. China Economic J. (2012) 5:131–49. doi: 10.1080/17538963.2013.764677, PMID: 24443653 PMC3893304

[15] Lin XR Zheng C Du ML Sun JH Liu YQ Zhao Z. Analysis of the health status and influencing factors of the elderly in China——based on the data of the 8th round of CLHLS data. Acad J Nav Medi Univ. (2022) 43:1022–8. doi: 10.16781/j.CN31-2187/R.20220149

[16] Ruel MT. Opereasonableizing dietary diversity: a review of measurement issues and research priorities. J Nutr. (2003) 133:3911S–26S. doi: 10.1093/jn/133.11.3911S 14672290

[17] Yin Z Fei Z Qiu C Brasher MS Kraus VB Zhao W. Dietary diversity and cognitive function among elderly people: a population-based study. J Nutr Health Aging. (2017) 21:1089–94. doi: 10.1007/s12603-017-0912-5, PMID: 29188865 PMC5726290

[18] Wang YF Tang Z Guo J Tao LX Liu L Li HB. BMI and BMI changes to all-cause mortality among the elderly in Beijing: a 20-year cohort study. Biomed Environ Sci. (2017) 30:79–87. doi: 10.3967/bes2017.011, PMID: 28292345

[19] Cheng FW Gao X Mitchell DC Wood C Still CD Rolston D. Body mass index and all-cause mortality among older adults. Obesity. (2016) 24:2232–9. doi: 10.1002/oby.21612 27570944

[20] Wang T Liang Y Wang YN Zhong YQ. Analysis of cognitive function change trajectory in middle-aged and elderly people based on latent variable growth mixed model. China J Chronic Dis Prev Control. (2022) 30:801–5. doi: 10.16386/j.cjpccd.issn.1004-6194.2022.11.001

[21] Duan X Dang Y Kang C Rong P Yan M Zhang S. Associations between trajectories of cardiovascular risk factor change and cognitive impairment in Chinese elderly: a nationwide cohort study. Front Aging Neurosci. (2023) 15:1084136. doi: 10.3389/fnagi.2023.1084136, PMID: 36845661 PMC9950264

[22] Dietary guidelines for adults with diabetes mellitus (2023 edition). Gen Pract Clin Educ. (2023) 21:388–91. doi: 10.13558/j.cnki.issn1672-3686.2023.005.002

[23] Chinese General Practice. Dietary guidelines for adults with hypertension (2023 edition). Gen Pract Clin Educ. (2023) 21:484–5. doi: 10.13558/j.cnki.issn1672-3686.2023.006.002

[24] Paulene C Kristin A Dara S. Sociodemographic and marital differences in spousal involvement in a partner^,^ s diabetes diet. Innov Aging. (2020) 4:453–3. doi: 10.1093/geroni/igaa057.1467

[25] Zhao XH Li JX. The impact of widowed on the health of the elderly in China: the moderating effect of social connection. J Demogr. (2022) 44:58–75. doi: 10.16405/j.cnki.1004-129X.2022.01.005

[26] Heuberger R Wong H. The association between depression and widowhood and nutritional status in older adults. Geriatr Nurs. (2014) 35:428–33. doi: 10.1016/j.gerinurse.2014.06.011 25085716

[27] Zisook S Shuchter SR Mulvihill M. Alcohol, cigarette, and medication use during the first year of widowhood. Psychiatr Ann. (1990) 20:318–26. doi: 10.3928/0048-5713-19900601-09

[28] Feraco A Armani A Amoah I Guseva E Camajani E Gorini S. Assessing gender differences in food preferences and physical activity: a population-based survey. Front Nutr. (2024) 11:1348456. doi: 10.3389/fnut.2024.1348456, PMID: 38445208 PMC10912473

[29] Razaz JM Balam FH Karimi T Rahmani J Kalantari N Shariatpanahi SP. Sex differences in healthy eating: investigating the moderating effect of self-efficacy. J Nutr Educ Behav. (2022) 54:151–8. doi: 10.1016/j.jneb.2021.05.011, PMID: 35148870

[30] Ter Bogt NCW Bemelmans WJ Beltman FW Broer J Smit AJ van der Meer K. Preventing weight gain: one-year results of a randomized lifestyle intervention. Am J Prev Med. (2009) 37:270–7. doi: 10.1016/j.amepre.2009.06.011, PMID: 19765497

[31] Lu X Zhang Q. Non-drug nutritional intervention for weight management in overweight and obese elderly. China Health Eng. (2015) 14:162–6. doi: 10.19937/j.issn.1671-4199.2015.02.021

[32] Zhang JD Gou B Wei W Peng W Feng X. 20 actions for weight loss in healthy weight management of Chinese residents: expert consensus based on scientific evidence. Chin J Diabetes Mellitus. (2023) 31:881–888.

[33] Senbanjo AFM Ottun TA. Early pregnancy body mass index, gestational weight gain and perinatal outcome in an obstetric population in Lagos, Nigeria. Pan Afr Med J. (2021) 39:136. doi: 10.11604/pamj.2021.39.136.25926 34527152 PMC8418156

[34] Liu M Zhang M Zhou J Song N Zhang L. Research on the healthy life expectancy of older adult individuals in China based on intrinsic capacity health standards and social stratification analysis. Front Public Health. (2024) 11:1303467. doi: 10.3389/fpubh.2023.1303467, PMID: 38356656 PMC10865369

[35] Wen FH Jiang YT. The logic and practice of sustainable spatial production for urban renewal finance. J West Hum Settlements. (2024) 39:14–21. doi: 10.13791/j.cnki.hsfwest.20240103

[36] Gu L Wang X Tian D. The association of family doctor contract service and patient trust in doctor: evidence from twenty-five village clinics of three counties in rural China. BMC Prim Care. (2024) 25:58–8. doi: 10.1186/s12875-024-02298-4 38360559 PMC10867991

